# Mapping the Mechanical Properties of Poly(3-hydroxybutyrate-co-3-hydroxyvalerate) Banded Spherulites by Nanoindentation

**DOI:** 10.3390/polym8100358

**Published:** 2016-10-12

**Authors:** Patricia Enrique-Jimenez, Juan F. Vega, Javier Martínez-Salazar, Fernando Ania, Araceli Flores

**Affiliations:** Department of Macromolecular Physics, Institute for the Structure of Matter (IEM-CSIC), Serrano 119 and 113bis, 28006 Madrid, Spain; patricia.enrique@csic.es (P.E.-J.); imtv477@iem.cfmac.csic.es (J.F.V.); j.msalazar@csic.es (J.M.-S.); f.ania@csic.es (F.A.)

**Keywords:** banded spherulites, poly(3-hydroxybutyrate-co-3-hydroxyvalerate), dynamic nanoindentation, AFM, lamellae twisting

## Abstract

Nanoindentation provides clear evidence that spherulite banding can be associated with a continuous modulation of mechanical properties from the more compliant peaks to the stiffer valleys. The structural arrangement in polymer-banded spherulites has intrigued scientists for many decades, and the debate has been recently intensified with the advent of new experimental evidence. The present paper approaches this issue by exploring the local mechanical properties of poly(3-hydroxybutyrate-co-3-hydroxyvalerate)-ringed spherulites via nanoindentation and discussing the confidence of the results. It was found that storage modulus and hardness across the banding morphology can be described as a sequence of regular oscillations with a periodicity that exactly matches the one observed using optical and atomic force microscopy. Results are consistent with the model of regular twisting of the lamellae, with flat-on arrangement in the low regions and edge-on lamellae in the crests.

## 1. Introduction

The structural arrangement giving rise to ring-banded spherulites has prompted an ongoing and intense debate [1,2,3,4,5,6]. A regular twisting of the lamellae from the center to the periphery was soon argued by Keller [7] and later formulated by Keith and Padden on account of uneven surface stresses in opposite lamellae fold surfaces [8,9,10]. Schultz and Kinloch described a model of giant dislocations giving rise to the twisting of the crystals [11], and Bassett and Hodge reported electron microscopy observations in which lamellar orientation was proposed to be assisted by the interleaving of untwisted lamellae [12]. A number of additional models have been postulated to account for lamellar rotation, all of them comprehensively described in fundamental reviews [6,13]. Those relying on structural factors, such as unbalanced surfaces stresses in opposite basal surfaces and the occurrence of screw dislocations, seem to be more generally accepted than other models based on distinctive features appearing on the growth front. The development of modern microdiffraction techniques allowed for the spatial resolution of the unit cell along a banded spherulite of poly(3-hydroxybutyrate) (PHB) at different locations by means of microbeam wide-angle X-ray diffraction (WAXS) [14,15,16]. The authors were able to conclude that the banding observed by polarized optical microscopy matched the radial distance between similar WAXS patterns [14,16]. Most importantly, a regular rotation of the PHB unit cell was observed, in agreement with a continuous torsion of the lamellae along the radius direction [15,16]. Moreover, high resolution real-time atomic force microscopy (AFM) observations on random copolymers of PHB and 3-hydroxyhexanoate provide clear evidence of individual lamella continuously twisting at the growth front [17]. Recent polarized infrared microspectroscopy studies on PHB have related a periodic oscillation of intensity of specific bands to variations in chain orientation and have also suggested a twisting of the lamellae in the radial direction [18,19].

The debate has been intensified in the last years with the release of new surveys on the subject [5,6]. On the one hand, a number of studies suggest discontinuity between the lamellar orientations of adjacent bands [5,20]. For example, observations on the interior morphology of blends of poly(ethylene oxide) (PEO) with poly(lactic acid) (PLA) and of poly(3-hydroxybutyrate-co-3-hydroxyvalerate) (P3HB-co-3HV) with poly(vinyl acetate) (PVAc) by means of scanning electron microscopy (SEM) suggest that a main lamellar orientation appears on one band, while lamellae are assembled as side branches on the adjacent band. In addition, a number of experimental evidences seem to be apparently at variance with the lamellar twisting model, such as the occurrence of circular cracks at the borders of the ridge and valley bands, or the asymmetry in the bands width [5]. Moreover, Woo and co-workers have demonstrated that banded spherulites can be generated in poly(1,6-hexamethyleneadipate) by alternating positive-negative birefringence rings using step crystallization [21]. It is noteworthy that lamellar twisting may not be a necessary condition for ring banding, but its occurrence would be manifested in a concentric banded morphology. On the other hand, the review by Crist and Schultz collects vast evidence by means of microscopic techniques of a continuous rotation of the lamellae associated with the optical banding on a variety of materials [6]. In particular, transmission electron microscopy observations show helicoidal twist of individual polymer ribbons and AFM images on polyethylene (PE) reveal uniform lamellar rotation along the radial direction with edge-on arrangement in the high regions and on the flat-on terraces in the low valleys. However, the same review also offers a few examples of a discontinuous change of lamellar orientation, including PE. More than fifty years after Keller’s first proposal of a regular lamellar twisting as the origin of polymer-banded spherulites, the issue remains open to hot debate.

Nanoindentation represents a powerful technique for the determination of the local mechanical properties of polymer materials. This can be accomplished using a purpose-designed instrumented nanoindenter or employing an atomic force microscope (AFM) as a nanoindentation testing device. The latter can give rise to a finer spatial resolution due to enhanced force and displacement sensitivity; however, calibration of the cantilever stiffness and of the tip contact area can still be an arduous task [22,23]. Alternatively, instrumented indentation also known as depth sensing indentation (DSI) constitutes a robust technique with well-established calibration methods that provide reliable mechanical properties. The most common analysis of the load-depth profiles in instrumented indentation assumes pure elastic behavior at the beginning of the unloading cycle [24,25]. The time-dependent behavior of polymers and soft materials has been a major concern among the indention community and specific strategies to extract meaningful information from DSI have been adopted [26,27,28,29]. For example, Tan and Ngan have proposed a rate-jump method that allows for the capture of a pure elastic response by eliminating the viscous component [29]. Scaling and dimensional analyses have also been shown to be most helpful for the understanding of the effects of the experimental conditions on the indentation response [28]. Significant work has been devoted to the development or adoption of mechanical models that can describe the indentation response [30,31,32,33,34,35,36], and linear viscoelasticity has been commonly assumed [30,31,32,33,34]. Power-law creep is also widely recognized as a valuable tool to analyze indentation data in terms of time-dependent plasticity [37]. Alternatively, dynamic DSI appears as a route to explore the time-dependent mechanical behavior. A small oscillation is superimposed to the semi-static force, allowing for a continuous measurement of the stiffness and damping of the contact and, in turn, providing complex modulus values as a function of penetration depth [25,38].

Thin films, coatings, polymer blends, or polymer composites are only some examples in which nanoindentation is most valuable for the characterization and comprehensive understanding of the material properties [39,40]. By tuning the indentation size, DSI can resolve the mechanical properties of a polymer material across the specimen thickness or along the surface providing fundamental information on morphological or nanostructural heterogeneities. It was soon realized that indentation can also distinguish between different crystal orientations in a polymer lamella [41]. In fact, early work on a ringed spherulite in blends of linear low density polyethylene (LLDPE) and high density polyethylene (HDPE) using AFM nanoindentation pointed towards a mechanical periodicity along the spherulite radius and a higher resistance to deformation in the valleys than on the peaks [39].

The present paper makes use of dynamic nanoindentation techniques to spatially resolve the properties across the banded morphology of a polymer spherulite. The aim is to offer new information based on the measurement of the local mechanical properties that can contribute to building an overall picture of the lamellar arrangement in ringed spherulites. We are confronted with the challenge of distinguishing whether the banding observed by optical microscopy and atomic force microscopy is associated with a concurrent change of mechanical properties. A critical discussion on a number of issues that may introduce uncertainties in the determination of mechanical properties by nanoindentation is offered. A P3HB-co-3HV copolymer has been selected due to the ease of band formation in a wide range of crystallization temperatures [42] and to the fact that the crystallization process, spherulite morphology and crystal structure have been thoroughly investigated [43,44,45].

## 2. Materials and Methods

The P3HB-co-3HV copolymer with 12 mol % HV by mol was purchased in powder form from Sigma-Aldrich Química S.L., Tres Cantos, Spain (Ref. 403121). It is a biodegradable, nontoxic, biocompatible plastic produced naturally by bacteria. Differential scanning calorimetry measurements (DSC7, Perkin-Elmer, Waltham, MA, USA) at 10 °C/min reveal a melting point of *T*_m_ = 155 °C. The raw material was melted at 175 °C, gently pressed between two glass plates for 2 min and subsequently crystallized at *T*_c_ = 60–65 °C for 2 h in a Mettler Toledo hot stage. The films have a final thickness in the range 8–150 µm.

AFM analysis was performed with a µTA™ 2990 Micro-Thermal Analyzer (TA Instruments, Inc., New Castle, DE, USA). The instrument was calibrated for displacement using a Topometrix silicon oxide-silicon grid. Topography maps were obtained in contact mode with a current set point of −2 nA that optimized the signal-to-noise ratio. A feedback loop keeps the cantilever deflection constant at the selected set point value while scanning. This is done by applying a voltage to the *Z*-piezo that was kept constant at 50 V. A V-shaped silicon nitride probe with a cantilever length of 200 µm and a spring constant of 0.032 N·m^−1^ was used. Depending on the size of the images (between 20 and 100 μm^2^), the scanning rates varied from 20 to 100 μm·s^−1^. The topography images were processed using the µTALab 1.01 software package provided with the system. Due to the instrumental limitations on the mounting of specimens on the sample holder, the height values for topography could be biased by the specimens tilt. This tilting effect was corrected by applying a software leveling function, fitting a surface to the observed topography and then subtracting the height values of the fitted surface pixel by pixel from those of the initial image. In this study, a first order plane was used. After leveling, a line analysis function, included in the software, was used to obtain the roughness of the observed specimens. 

Nanoindentation, or depth-sensing indentation (DSI), tests were carried out using a G200 Nanoindenter (Keysight Tech., Santa Rosa, CA, USA) with a continuous stiffness measurement (CSM) option and a low load resolution head (dynamic contact module, DCM). A Berkovich diamond indenter with a tip radius lower than 20 nm was employed, and its tip area function was calibrated against a fused silica standard following the method described in [25]. During the loading cycle, a constant indentation strain rate of 0.05 s^−1^ was selected. A small oscillating force of 2 nm at a frequency of 75 Hz was superimposed to the quasi-static loading. The contact stiffness at any point during the loading portion is determined from the phase lag between the oscillating force and the indenter penetration produced, following the procedure described in detail in [25,38,46,47]. In turn, storage modulus and hardness can be determined assuming elastic-viscoelastic correspondence [38,48]. The projected contact area is calculated on the basis of the contact depth using the Oliver–Pharr approach [25].

## 3. Results

### 3.1. Local Mechanical Properties across the Banding Morphology

**Figure 1:**
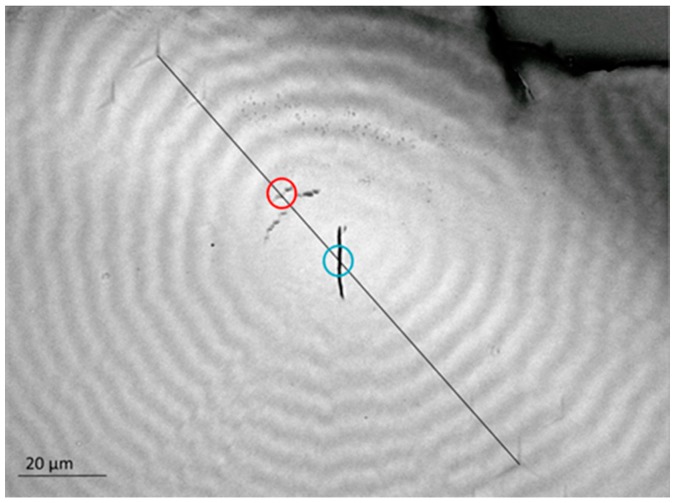
Optical microscopy image of the banded morphology of P3HB-co-3HV after crystallization at 60 °C. Indentations of *h* ≈ 100 nm separated by 1 μm were produced along the distinct line. Circles identify two cracks appearing across the straight line, the blue one denoting the crack in the center of the spherulite.

**Figure 2:**
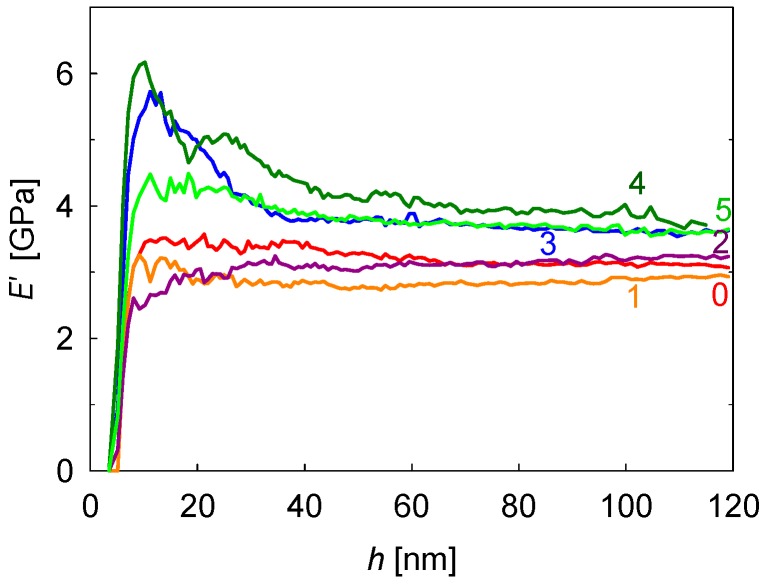
Storage modulus, *E*′, measured as a function of penetration depth, *h,* for the first six indentations of the scanning line of Figure 1. Numbers indicate the distance (in µm) from each indent to the starting point.

Figure 1 shows a well-developed banded morphology appearing after the crystallization of P3HB-co-3HV at 60 °C. The band spacing of the spherulite shown in Figure 1 as observed by optical microscopy (OM) is 6–7 μm. Due to the brittle nature of the material, the sample showed the occurrence of cracks, most frequently located at the center of the spherulites. The mechanical properties across the banded morphology were explored by producing 120 indentations along a straight line using a maximum penetration depth *h* ≈ 100 nm. The size of the indentations along the distinct line of Figure 1 is beyond the resolution of OM. In contrast, post-test large indentations can be clearly distinguished and were produced to identify the beginning (top left) and the end (bottom right) of the scanning line. The separation between indents along this line was selected to be 1 µm. Taking into account the geometry of the Berkovich indenter (*l* = 6.52 × *h*, where *l* is the height of the projected triangular indent), the distance between the apex of one indent to the side of the next one is 350 nm; hence, overlapping was avoided. The spacing between indents was carefully selected to achieve the necessary spatial resolution across the band morphology and at the same time a negligible interaction between the deformation fields of neighbouring indents. The latter was confirmed by the fact that the mechanical property remains constant as a function of indentation depth (see Figure 2).

**Figure 3:**
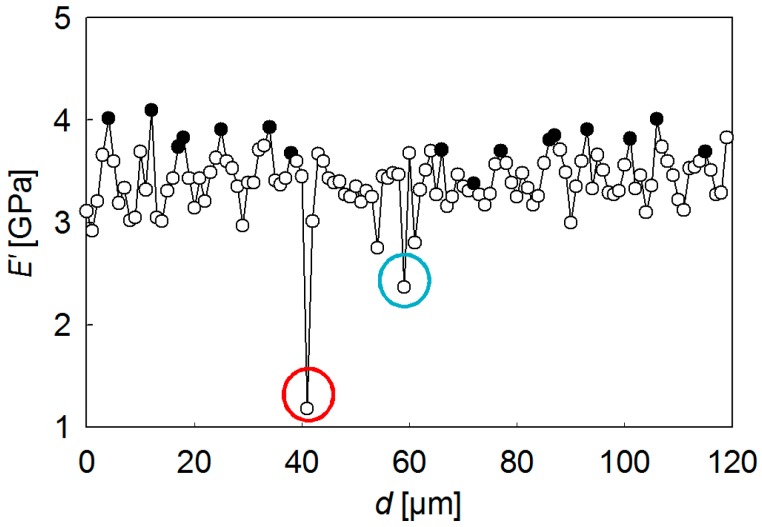
Plot of *E*′ (for *h* = 100 nm) as a function of the distance *d* to the starting point of the scanning line of Figure 1. Drawn circles denote the location of cracks.

Figure 2 shows the *E*′ behavior as a function of indenter penetration depth, *h*, for the first six indents along the scanning line of Figure 1. A color code has been used to differentiate the profiles arising at different locations. Numbers in Figure 2 indicate the distance (in µm) from each indent to the first one in the line. It should be noted that the strong variation of *E*′ data with a penetration depth for *h* < 30 nm cannot be taken into consideration because it is associated with a number of factors including the inaccurate calibration of the tip area at these small penetration depths, the transition from the deformation mode of a sphere (due to tip rounding) to a pyramid, roughness effects, etc. [40]. All these issues can be disregarded for *h* > 30 nm, where the *E*′ values approach a constant value that should be representative of each specific location. It was found that *E*′ for indents at 0, 1, and 2 µm lies in the range of 2.9–3.2 GPa, while the *E*′ values move towards ≈4 GPa for indents at 3, 4, and 5 µm. This significant difference (≈30%) suggests that a variation in the mechanical property across the band morphology takes place. Indeed, Figure 3 shows the plot of *E*′ at *h* = 100 nm, as a function of the distance to the first indentation of the scanning line, *d*. The two lowest *E*′ values in the plot (indicated by a blue and a red circle) can be associated with the occurrence of cracks. The rest of the *E*′ profile represents a well-defined sequence of peaks and valleys with a periodicity of 6–7 μm. Solid symbols correspond to indentations initiated on a dark band in the OM image of Figure 1. It is clearly seen that the highest *E*′ values are associated with the dark bands, while the lowest are associated with the light regions. *E*′ average values for mechanical crests and valleys are 3.8 ± 0.2 GPa and 3.1 ± 0.1 GPa, respectively.

Figure 3 addresses three important issues: (i) the mechanical periodicity matches the optical microscopy pitch; (ii) a continuous modulation of *E*′ across the spherulite banding, and not discontinuous changes, takes place; (iii) the *E*′ values are maximum in the dark bands and minimum in the bright ones. It should be noted that flat-on arrangement is expected to yield higher mechanical properties than edge-on orientation [41,49]. Therefore, our experimental results are in agreement with preceding AFM findings showing flat-on lamellae in the valleys corresponding to the polarized OM dark rings and edge-on arrangement at the ridges associated with the bright areas [6,50].

At this point, it is worth discussing the fact that the mechanical property at each location represents the average mechanical property of a whole volume of deformation underneath the indenter subjected to a stress field that evolves radially from the point of initial contact. Thus, one could call into question whether the gradual variation shown in Figure 3 (and Figure S1) could arise from a step-wise change in mechanical property. A detailed analysis of Figure 2 shows that, in all cases, *E*′ values remain constant from *h* ≈ 30 nm up to *h* ≈ 100 nm; while this could never be explained considering a sharp change between hard and soft bands, it could be well compatible with a continuous variation of mechanical properties.

### 3.2. Mapping a Spherulite

**Figure 4:**
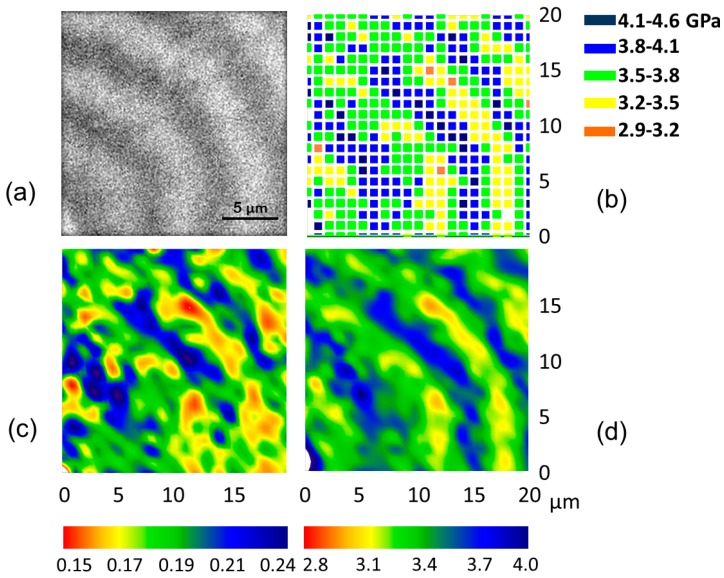
(**a**) Optical micrograph of the area probed using an array of 21 × 21 indentations separated by 1 µm. The spherulite center is located at (0,0); (**b**) Mesh of indentation modulus values at specific *X* and *Y* positions. The color code appears on the right-hand side of the map; (**c**) *H* and (**d**) *E*′ contours, respectively, constructed by interpolating the mesh of indentation data. The color scale (in GPa) appears at the bottom of the respective plots.

The modulation of mechanical properties along the spherulite radius has been further explored by mapping the mechanical properties of P3HB-co-3HV spherulites over selected areas. Indentation arrays of 21 × 21 indentations with a *h* of 100 nm separated by 1 μm and covering an area of 20 × 20 µm^2^ were produced. Figure 4a shows the optical micrograph of one of the regions selected for nanoindentation. Several bright and dark bands can be discerned, the spherulite center being at the (0,0) coordinate.

Figure 4b illustrates the plot of the storage modulus values represented using a color code at each *X* and *Y* position. It is remarkable how the regions with the highest *E*′ values (blue symbols) reproduce the shape of the dark rings appearing in the optical micrograph. Similarly, the regions of the minimum *E*′ values outstandingly define the bright bands of Figure 4a. The fact that the *E*′ values gradually increase and decrease across the spherulite banding with the same periodicity as the optical pitch is also interesting. Hardness values, *H*, present a similar behavior, and results are not presented for the sake of simplicity. Figure 4c,d illustrate the contour plots arising from the interpolation of the raw *H* and *E*′ data, respectively.

### 3.3. Correspondence between AFM (Atomic Force Microscopy), OM (Optical Microscopy), and Nanoindentation Results

**Figure 5:**
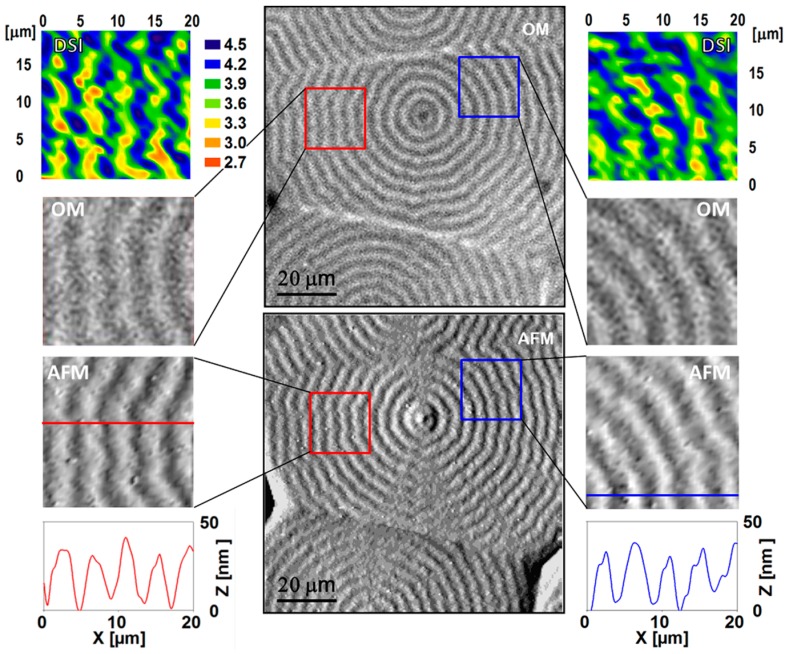
Central part: P3HB-co-3HV spherulites grown after crystallization at *T* = 65 °C. The images were taken using OM (top) and AFM (bottom). Enlargements of the two areas selected for analysis (20 × 20 μm^2^) are shown on the left- and right-hand sides, together with the *E*′ contours constructed by interpolating the mesh of indentation data (top) and the AFM height profiles (bottom) across the lines indicated in the enlarged AFM images. The *E*′ color code appearing next to the contour map on the left-hand side has also been used for the contour map on the right-hand side (units in GPa).

Selected areas of interest were investigated using OM, AFM, and nanoindentation. In order to localize the same region by the three techniques, a well-defined pattern of big square indents (diagonal ≈ 50 µm) was produced with the help of a Vickers microindenter to delimit the area of interest. This pattern was used as a coordinate system for the selected regions. Figure 5 (central part) shows a general view of the sample surface, as obtained via OM (top) and AFM (bottom). Nanoindentation experiments on two different locations, marked in Figure 5 with a red and a blue square, are included. Grids of 21 × 21 indentations with an *h* of 100 nm separated by 1 μm were produced. Nanoindentation, OM, and AFM results for each one of these selected areas are included in Figure 5 on the left- and right-hand sides. The AFM profiles included at the bottom of the figure show a sequence of uniform and regular oscillations with a periodicity of ≈4.5 µm and a peak-to-valley height of ≈30–40 nm. It is clearly seen that the AFM periodicities exactly match the optical microscopy pitches. Moreover, AFM images confirm that the peaks and valleys can be associated with the bright and dark rings observed via optical microscopy, respectively. Concerning nanoindentation, it is found that the mapping of the mechanical properties outstandingly reproduces the ringed morphology and that the variation of *E*′ across the spherulite banding exhibits the same regularity as the AFM and the OM images.

## 4. Discussion

Results shown in Section 3 suggest that a continuous modulation of the mechanical properties takes place across the banding morphology and that this correlates with the peaks and valleys observed via optical microscopy and AFM. It is of great importance to assess that this *E*′ and *H* gradual variation is not affected by factors inherent to the indentation process or to the data analysis; therefore, a number of issues are discussed in what follows.

### 4.1. Tip Calibration

The procedure employed in the present paper to derive *E*′ and *H* requires an estimation of the contact area between the indenter and the sample, and this involves a calibration of the tip area that has been accomplished using fused silica, as described in the experimental section [25]. It has been suggested that the contact between a diamond indenter and a hard surface like silica may not resemble the interaction of the same tip with softer materials; hence, calibration of the area function against a polymer reference could be more appropriate [26,51]. One way to account for the interaction between the indenter and the polymer is to introduce an “apparent tip defect” [51], but the extent of this “apparent tip defect” has been shown to significantly depend on the specific polymer material being tested [52]. Hence, even in the case where a polymer reference was used, the calibrated contact area can introduce some uncertainty on the *E*′ and *H* values. Consequently, in the present paper, we have adopted the following criteria: the indenter area function is calibrated against silica and the mechanical behavior is substantiated by inspection of the contact stiffness *S*, which is a property determined from the phase lag between oscillation force and displacement and does not require figuring out the contact area [25,38,46,47]. Figure S1 of the Supplementary Materials illustrates the *S* variation as a function of the distance to the first indentation in the scanning line of Figure 1, and Figure S2 shows the *S* contour plot of the region of Figure 3. It is clearly seen that, similarly to *E*′ and *H*, the *S* behavior is described by a periodic sequence of peaks and valleys across the spherulite radius, and this strongly supports the occurrence of a continuous modulation of mechanical properties.

### 4.2. Pile-Up Behavior

**Figure 6:**
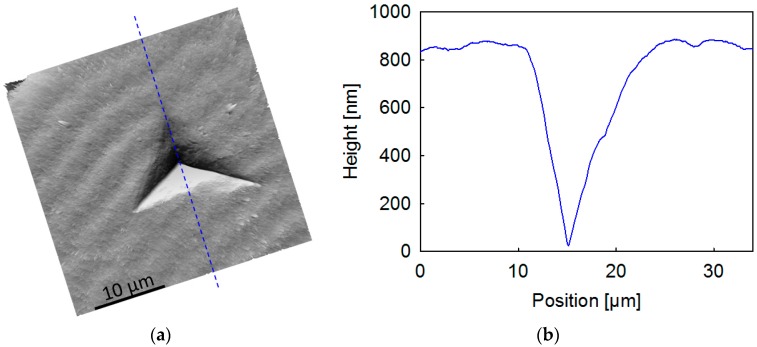
(**a**) AFM image of an indentation produced on the surface of the P3HB-co-3HV spherulite of Figure 5; (**b**) Profile along the blue line marked on (**a**).

Pile-up around the indents represents an issue of major concern that can produce an increase in the *E*′ and *H* values as high as 50% [53]. This is because the “true” area of contact between the indenter, and the material is higher than that taken into account for the calculations. Pile-up behavior is commonly associated with materials with limited capacity to work-harden and a large *E*/*H* ratio (more than 100) [28,53,54]. It has been suggested that the *h*_f_/*h*_max_ (or similarly *h*_c_/*h*_max_) experimental ratio describes the pile-up behavior [28,53], where *h*_max_ is the maximum penetration depth during the loading cycle, *h*_c_ is the contact depth, and *h*_f_ is the final depth after load removal. Finite element simulations suggest that significant pile-up develops close to 1 for *h*_f_/*h*_max_ and can be disregarded for *h*_f_/*h*_max_ < 0.7. In the present case, *E*/*H* << 100; in fact, this ratio is always below 30. On the other hand, inspection of the load-unloading curves of indentations produced on P3HB-co-3HV spherulites reveals *h*_f_/*h*_max_ ≈ 0.75 (see Figure S3 of the Supplementary Materials), and this value is not conclusive to disregard pile-up effects. Hence, direct observation of an indent was carried out using AFM. To better assess pile-up behavior, we have produced large indentations where the effect is expected to be more important [55]. Figure 6 illustrates the AFM image of one indentation produced on the surface of the P3HB-co-3HV spherulite of Figure 5. Figure 6 also illustrates the profile along the line marked in the AFM image. It is clearly seen that there is no significant protruded material around the indent. The periodic hills and valleys at both sides of the indent profile are associated with the waviness of the spherulite banding. On the other hand, there is also rather limited sinking-in, and this should be accounted for in the derivation of the contact depth as calculated with the Oliver–Pharr method [25].

### 4.3. Surface Topography

During the revision process of the manuscript, the question about the possible influence of sample topography on the nanomechanical data recorded was raised. It is evident from the AFM height profiles shown in Figure 5 that the spherulite structure produces surface undulations, and the indenter tip is in fact indenting on surfaces with different slopes. However, the different scale used for both axes in the profiles should be noticed; while the height (*Z* axis) is expressed in nm, the position is given in µm (*X* axis). Differences in height are around 50 nm, and they are periodically separated by a distance of 4–5 µm. The maximum slope angle measured is less than 1.5°. Although this figure does not seem to be relevant, a general discussion on the changes that different surface slopes can produce on nanoindentation modulus and hardness seems very attractive. To the best of our knowledge, it has not been handled before.

**Figure 7:**
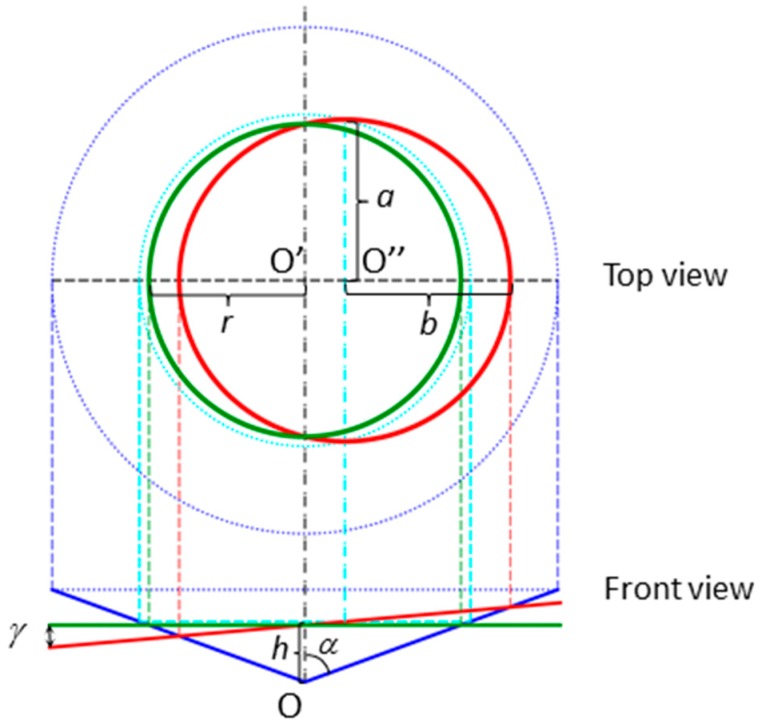
Front view: Graphical representation of a right circular conical indenter penetrating a normal (green solid line) and an oblique surface with *γ* = 5° (red solid line). Top view (rotated 90°): corresponding projected contact areas with the same color code. Cyan dotted lines represent the circular straight cross-section of the cone passing through the center O″ of the oblique red ellipse. Their intersection provides the semi-axis *a*. All vertical dotted lines are imaginary projection lines.

To simplify the problem and obtain an analytical expression for the projected contact area, the pyramidal indenter is replaced by an equivalent right circular cone with a cone angle giving the same area-to-depth ratio [56]. In our case, the Berkovich indenter can be replaced by a conical one with a half-apical angle *α* of around 70°. Figure 7 graphically summarizes the discussion, and it should be emphasized that it is only based in geometrical considerations. The front view at the bottom of the figure shows a conical indenter (half angle *α*) intersected by two planes. The green one represents a perpendicularly indented surface, while the red surface has a constant slope angle *γ*. In both cases, the penetration depth is *h*. The intersection of a plane with a right circular cone, whose axis OO’ is perpendicular to the plane, produces a circle. The projection of this circle or projected contact area is the circle limited by the green circumference shown in the top view. The center O’ of the circle lies on the OO’ axis of the cone. On the other hand, an indentation on a surface with a slope angle *γ* < *α* can be visualized as the intersection of an oblique plane with a cone and yields an ellipse, whose projection onto a normal plane is again an ellipse cantered in O’’ (drawn in red in the top view of the figure).

The radius *r* of the circle and the semi-axes *a* and *b* of the ellipse can be readily calculated as a function of penetration depth *h*, semi-apical cone angle *α*, and slope angle *γ*.

(1) r=htanα

(2) $$a=\frac{h\;\tan^{-1}\alpha }{\tan^{-2}\alpha -\tan^{2}\gamma }$$

(3) $$b={\left[a^{2}-\frac{{\left(a\;\tan^{-1}\alpha -h\right)}^{2}}{\tan^{2}\gamma }\right]}^{1/2}$$

Then, the relative variation of the projected contact area of an indentation produced on a surface at an angle *γ* in relation to a normal indentation is the difference between the area of the red ellipse and the green circle, divided by the area of the circle. This means that

(4) $$\Delta A=\frac{\pi ab-\pi r^{2}}{\pi r^{2}}=\frac{ab}{r^{2}}-1$$

Now taking into account the well-known relationship between the projected contact area and the storage modulus *E*′ (Equation (6) in [53]), it turns out that the relative variation in modulus is given by

(5) $$\frac{\Delta E^{\prime }}{E^{\prime }}=\frac{\Delta A}{2A}$$

In a similar manner (Equation (5) in [53]), the relative variation in indentation hardness is

(6) $$\frac{\Delta H}{H}=\frac{\Delta A}{A}$$

The above equations allow for the estimation of the relative error in the experimental values of the nanoindentation storage modulus and hardness obtained on a surface with a certain slope angle and for different conical indenter geometries. The values affected by a slope should always be higher than the normal ones because the projected contact areas of the ellipses are always higher than the area of the normal circle.

Figure S4 of the Supplementary Materials shows the relative error in modulus as a function of the slope angle *γ* for the particular case of a conical indenter that is equivalent to a Berkovich indenter (*α* ≈ 70°). It can be shown that this curve is independent of the penetration depth *h.* In our particular case of the undulated surface of a spherulite structure that presents maximum slope angles *γ* < 1.5°, the experimental values of both storage modulus and hardness can be overestimated by no more than 1%.

All of the above considerations point toward a genuine continuous modulation of mechanical properties across the spherulite banding from the stiffer valleys to the more compliant ridges. Higher *E*′ values in the valleys and lower ones on the crests are compatible with the occurrence of flat-on lamellae and edge-on arrangement, respectively, as suggested above. Results point toward the existence of a whole range of values that are intermediate between those associated with the crests and to the valleys, and in accordance with the model of a continuous twisting of the lamellae along the spherulite radius. It is remarkable that the mechanical periodicity has been likewise found for a range of film thicknesses, from 8 µm (Figure 5) up to relatively thick films of 150 µm (Figure 1). This implies that the surface molecular arrangement is independent of spherulite thickness for the investigated samples. One argument used against reports of continuous twisting measured by microbeam WAXS and polarized OM is that the information is obtained through the thickness of the specimens and thereby smooths out the signals from discontinuous entities. In the present work, the continuous change of mechanical properties across the spherulite banding arises from the direct feedback at the sample surface, and it has been shown that the measured continuity does not depend on the depth from which the signal is integrated, within a significant range of depths. This issue represents by itself an important advantage of nanoindentation with respect to the use of other techniques.

## 5. Conclusions

In conclusion, nanoindentation provides clear evidence that spherulite banding in P3HB-co-3HV is associated with a continuous change of mechanical properties from the more compliant peaks to the stiffer valleys. A periodical sequence of mechanical maxima and minima is found, in accordance with the flat-on arrangement of the lamellae in the valleys and edge-on in the ridges. It has been shown that this continuous modulation is not affected by factors inherent to the indentation process or to the indentation analysis, such as pile-up and surface topography. Concerning the latter, a general equation that allows for the estimation of the influence of the surface average slope on the projected contact area and, hence, on *E*′ and *H* is offered.

Moreover, the parallelism found between the local mechanical properties and the ringed morphology is noteworthy. It has been shown that the distance between adjacent mechanical maxima matches the regularity of the spherulite banding by OM, and both of them are in notable correspondence with the distance between adjacent valleys (or peaks) via AFM. This work was not intended to solve the controversy on all types of spherulites, as it may be the case that twisting is continuous in some systems, but not in others. Nevertheless, it offers clear and unambiguous results on banded spherulites of P3HB-co-3HV. In this particular case, the combined analysis of AFM, nanoindentation, and optical microscopy studies is in agreement with the generalized model of a continuous twisting of the lamellae along the spherulite radius. 

## Supplementary Materials

The following are available online at www.mdpi.com/2073-4360/8/10/358/s1, Figure S1: Plot of Stiffness *S* (for *h* = 100 nm) as a function of the distance *d* to the starting point of the scanning line of Figure 1; Figure S2: Stiffness contour constructed by interpolating the mesh of indentation data taken on the selected area of a P3HB-co-3HV spherulite shown in Figure 4a; Figure S3: Load-depth profile associated with the third indentation on the line of Figure 1, produced on the surface of a P3HB-co-3HV spherulite; Figure S4: Relative variation in indentation storage modulus *E*′ as a function of the slope angle that forms the oblique surface of the sample with a plane normal to the indentation direction.
