# Supplementary material for: Mapping the Mechanical Properties of Poly(3-hydroxybutyrate-co-3-hydroxyvalerate) Banded Spherulites by Nanoindentation
Source: Polymers (Basel). 2016 Oct 12;8(10):358. doi: 10.3390/polym8100358 (PMC6432475; doi:10.3390/polym8100358)
Supplement: Supplementary file 1 [file polymers-08-00358-s001.pdf]

Supplementary Materials: Mapping the Mechanical Properties of Poly(3-hydroxybutyrate-co-3-hydroxyvalerate) Banded Spherulites by Nanoindentation

Patricia Enrique-Jimenez, Juan F. Vega, Javier Martínez-Salazar, Fernando Ania and Araceli Flores

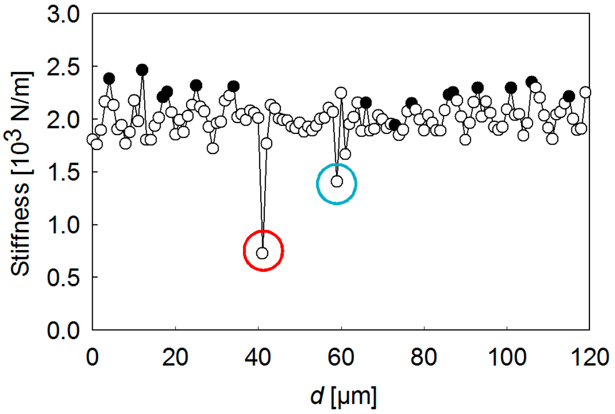

Figure S1. Plot of S (for h = 100 nm) as a function of the distance d to the starting point of the scanning line of Figure 1. Drawn circles denote the location of cracks.

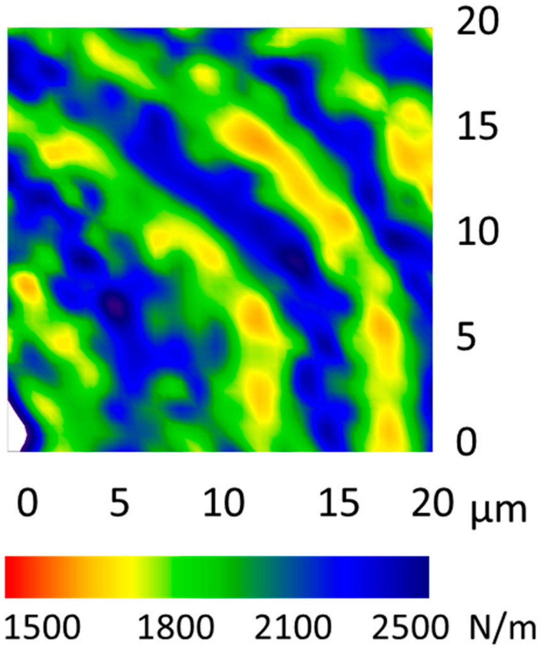

Figure S2. Stiffness contour constructed by interpolating the mesh of indentation data taken on the selected area of a P(3HB-co-3HV) spherulite shown in Figure 4a.

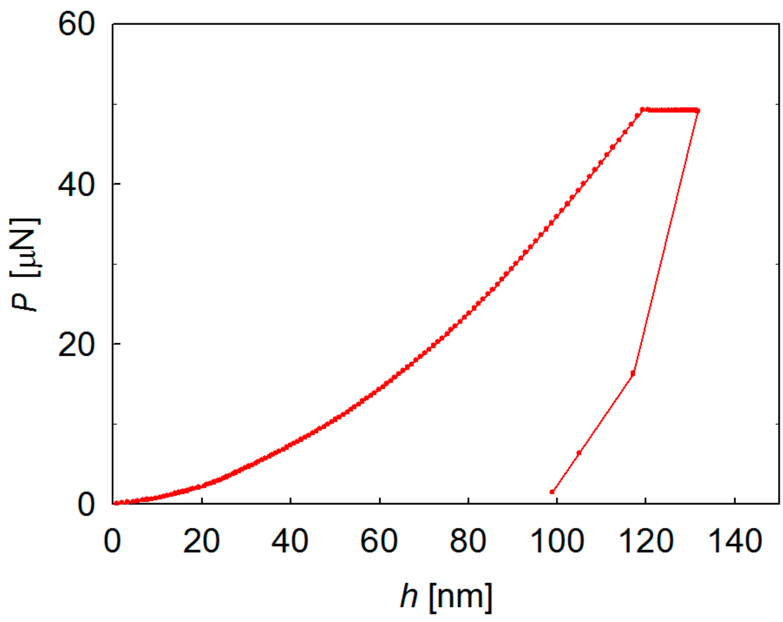

Figure S3. Load-depth profile associated to the third indentation on the line of Figure 1, produced on the surface of a (P3HB-co-3HV) spherulite.

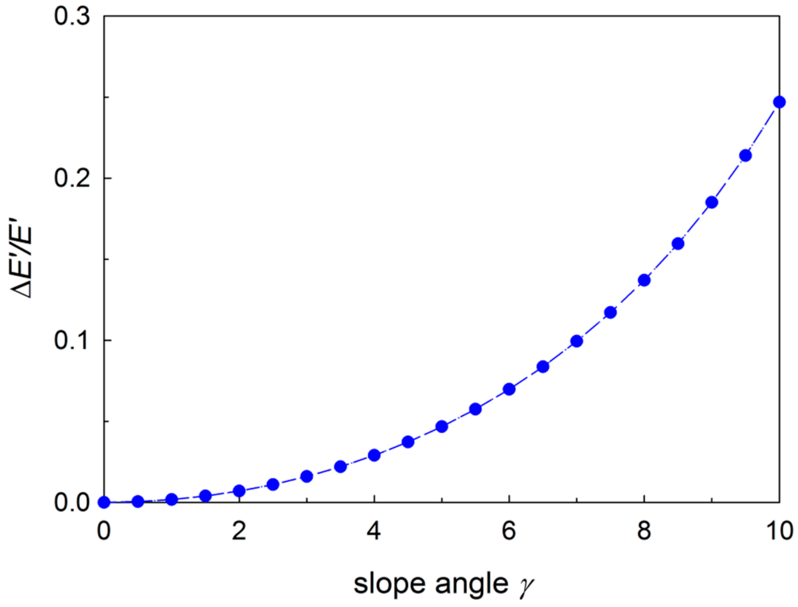

Figure S4. Relative variation in indentation storage modulus E' as a function of the slope angle that forms the oblique surface of the sample with a plane normal to the indentation direction.
